# Modality compatibility in task switching depends on processing codes and task demands

**DOI:** 10.1007/s00426-020-01412-2

**Published:** 2020-09-07

**Authors:** Erik Friedgen, Iring Koch, Denise Nadine Stephan

**Affiliations:** Institute of Psychology, RWTH Aachen University, Jägerstr. 17/19, 52066 Aachen, Germany

## Abstract

Modality compatibility denotes the match between sensory stimulus modality and the sensory modality of the anticipated response effect (for example, vocal responses usually lead to auditory effects, so that auditory–vocal stimulus–response mappings are modality-compatible, whereas visual–vocal mappings are modality incompatible). In task switching studies, it has been found that switching between two modality-incompatible mappings (auditory-manual and visual–vocal) resulted in higher switch costs than switching between two modality-compatible mappings (auditory–vocal and visual-manual). This finding suggests that with modality-incompatible mappings, the anticipation of the effect of each response primes the stimulus modality linked to the competing task, creating task confusion. In Experiment 1, we examined whether modality-compatibility effects in task switching are increased by strengthening the auditory–vocal coupling using spatial-verbal stimuli relative to spatial-location stimuli. In Experiment 2, we aimed at achieving the same goal by requiring temporal stimulus discrimination relative to spatial stimulus localisation. Results suggest that both spatial-verbal stimuli and temporal discrimination can increase modality-specific task interference through a variation of the strength of anticipation in the response-effect coupling. This provides further support for modality specificity of cognitive control processes in task switching.

When two tasks have to be performed in alternation, higher response time (RT) and error rates can be observed (see Kiesel et al., 2010; Koch, Poljac, Müller, & Kiesel, 2018, for reviews). Such costs do not only arise when switching from one task to the other (switch costs), but occur even when a task is repeated: The mere presence of another task in the same block of trials leads to worse performance compared to a block containing only one of the two tasks (mixing costs).

In research on task switching, switch costs are often understood as reflecting the competition of task representations (task sets) and the need to “reconfigure” the current task set (Monsell, 2003). Meanwhile, mixing costs have been interpreted as either reflecting the higher load on working memory that arises from having to maintain two task sets in an activated state rather than just one (e.g. Los, 1996), or, alternatively, to the uncertainty of which task will have to be performed next (e.g. Poljac, Koch, & Bekkering, 2009; Rubin & Meiran, 2005).

In dual-task research, such performance costs of multitasking have often been attributed to a structural bottleneck (Pashler, 1994) or to a shared, but content-free resource (Kahneman, 1973). In contrast, Wickens (1984, 2008) proposed a modality-specific model in which visual and auditory perception rely on different resources. Recent evidence has been hinting towards modality-specific influences on control processes in addition to central interference (e.g. Hazeltine, Ruthruff, & Remington, 2006; Schacherer & Hazeltine, 2020; see Koch et al., 2018, for a review). In the present study, we focus on modality-specific effects in task switching.

Stephan and Koch (2010) developed a spatial-discrimination paradigm to investigate modality-compatibility effects in task switching. Modality compatibility refers to the degree of similarity between the modality of the stimulus and the modality of the sensory consequences related to the response (Stephan & Koch, 2010). The concept of modality compatibility is based on the idea of ideomotor compatibility (Greenwald, 1972), which considers a stimulus and a sensory response effect compatible to the extent that they are similar to each other. This similarity, however, includes stimulus identity (for example, having to say “left” in response to hearing the word “left”), and is therefore more narrow than the idea of modality compatibility. In contrast, the setup of a consecutive study by Stephan and Koch (2011) where participants had to respond by saying “A” or “one” in response to the auditory stimuli “X” and “M” would be considered modality-compatible because the sensory modality of the response effect is in accordance with the modality of the stimulus (see also Schacherer & Hazeltine, 2019); however, it would not be ideomotor-compatible because stimulus identity and response identity were not the same.

It should be noted that the concept of ideomotor compatibility is based on the ideomotor principle (James, 1890), which states that actions are initiated on the basis of their anticipated effects (see also Hommel, Müsseler, Aschersleben, & Prinz, 2001), and recent evidence suggests people also monitor whether these action effects actually occur (Wirth, Janczyk, & Kunde, 2018; Wirth, Steinhauser, Janczyk, Steinhauser, & Kunde, 2018). Thus, throughout our lives, we experience that vocal responses usually lead to auditory effects, whereas manual actions tend to result in visible changes in the environment, such as when viewing the hand moving in the case of eye-hand coordination in grasping actions. Therefore, an auditory–vocal and a visual-manual stimulus–response mapping can be considered modality-compatible, relatively speaking, while the opposite mappings, that is, auditory-manual or visual–vocal, would be classified as less modality-compatible or, relatively, modality-incompatible (Stephan & Koch, 2011, 2015).

In their study, Stephan and Koch (2010) found that switch costs were larger with modality-incompatible mappings (visual–vocal and auditory-manual) than with modality-compatible mappings (visual-manual and auditory–vocal). To understand the origins of the influence of modality compatibility in task switching, first one needs to note that this increase in switch costs has only been found when switching between *two* modality-incompatible mappings compared to *two* modality-compatible mappings, suggesting that interference only arises with specific combinations of modality mappings, rather than with individual incompatible modality mappings per se (Fintor, Stephan, & Koch, 2018a). For example, in a modality-incompatible condition, performing a vocal response following a visual stimulus primes, by means of the anticipated action effect, the processing of auditory input, that is, the competing auditory-manual task, resulting in task confusion; the two task-sets interfere and create crosstalk (Stephan & Koch, 2011, 2015, 2016). If the stimulus is now manipulated to be even more similar to the anticipated response effect—for example, an auditory stimulus which is also a word (Schäffner, Koch, & Philipp, 2018), like the usually verbal output of a vocal response—this between-task confusion should become even stronger: A vocal response to a verbal stimulus would prime the processing of verbal auditory input, even if the stimulus instructing this vocal response is a visual stimulus. Likewise, an auditory verbal stimulus would prime a vocal response even more strongly; the added similarity to the anticipated effect of said response would further simplify the response-selection process (Schacherer & Hazeltine, 2020). Consequently, this priming should then also occur if the instruction actually requires a manual response to auditory stimuli. If both of these primes occur in the same block (i.e. a block using two incompatible modality mappings), crosstalk between these two tasks should be increased.

In the present study, we aimed at examining whether modality compatibility is affected by variations in the type of task (spatial stimulus localisation vs. temporal stimulus-duration discrimination) and the type of the processing code (spatial-location vs. spatial-verbal codes) in two experiments. In both experiments we asked for manual vs. vocal responses. Note that we also included single-task blocks to assess mixing costs in addition to switch costs in mixed-task blocks.

Our basic predictions could be related to the modality-specific resource model suggested by Wickens (1984, 2008), which distinguishes between spatial and verbal processing codes. Specifically, the model links manual responses to the spatial processing code and vocal responses to the verbal processing code. By extension, since manual responses are modality-compatible with visual stimuli and vocal responses are modality-compatible with auditory stimuli, Wickens, Vidulich and Sandrygarza (1984) also proposed a connection between the spatial processing code and visual perception, as well as between the verbal processing code and auditory perception, because verbal stimuli are usually encountered in the context of speech.

In the present Experiment 1, we tested whether the use of spatial-verbal stimulus material would strengthen the auditory–vocal coupling compared to stimuli referring to spatial location. To this end, we employed the spatial-location discrimination task employed by Stephan and Koch (2010), who used visual stimuli presented on the left or right side of the screen and auditory stimuli (tones) presented on the left or right ear, and compared the influence of modality compatibility on task switching with these spatial-location stimuli to that on a similar paradigm using stimuli with the spatial-verbal meaning “left” and “right.” We expected a larger influence of modality compatibility with spatial-verbal stimuli based on previous findings reported by Schäffner et al. (2018), who had already demonstrated larger effects of modality compatibility on switch costs with verbal stimuli compared to nonverbal stimuli (see also Göthe, Oberauer, & Kliegl, 2016, for related ideas in dual-task research). However, they examined the influence of verbal vs. spatial stimuli in a between-subjects design using semantic classification tasks; in contrast, we aimed at isolating, more specifically, the role of spatial-location stimuli vs. spatial-verbal stimuli in modality compatibility in task switching.

In Experiment 2, we considered another possibility to strengthen the auditory–vocal coupling, namely using a temporal-discrimination task compared to a spatial-discrimination task. In research on crossmodal attention, it has been found that spatial-discrimination tasks usually elicit visual dominance (e.g. Lukas, Philipp, & Koch, 2010; see also Spence, Parise, & Chen, 2012; for a review), whereas temporal-discrimination tasks often elicit auditory dominance (e.g. Lukas, Philipp, & Koch, 2014; Repp & Penel, 2002). Therefore, in Experiment 2, we compared modality compatibility effects with spatial-location stimuli with those in a temporal duration discrimination task, in which stimuli were either presented for a short or long time.

Increasing the strength of the coupling between a response modality and the modality of its anticipated effect should result in larger interference between mappings. Spatial-verbal processing codes and/or temporal-discrimination task demands should increase the auditory–vocal coupling compared to spatial-location stimuli and thus lead to a larger effect of modality compatibility on mixing costs and switch costs. Consequently, we expected an increased modality-compatibility effect on mixing costs and switch costs for spatial-verbal processing codes (Experiment 1) and temporal-duration task demands compared to spatial-location processing codes/task demands (Experiment 2).

## Experiment 1

In Experiment 1, we used a spatial-discrimination task with spatial-verbal and spatial-location stimuli in modality-compatible and in modality-incompatible stimulus–response mappings. The spatial relation between stimulus and response was always compatible (for example, “left” stimuli always call for “left” responses).

Schäffner et al. (2018) systematically combined verbal vs. non-verbal stimulus codes (that is, written/spoken words vs. pictures/sounds) and spatial vs. nominal response codes (that is, words which describe a location, like “left”/“right”, vs. a category/concept, like “insect” or “instrument”) with compatible and incompatible modality mappings. The authors found larger switch costs when switching between two incompatible modality mappings compared to switching between two compatible modality mappings, but these modality-compatibility effects were larger for verbal input codes compared to non-verbal input codes. Schäffner et al. (2018) attributed these findings to more pronounced links between verbal stimuli and verbal response effects. However, their verbal stimuli required semantic categorisation (into the categories “living” and “non-living”), and only the responses consisted of saying the words “left” and “right” or pressing left and right keys. In contrast, in our study, the dichotomy was spatial-location vs. spatial-verbal input codes, that is, the verbal stimuli still referred to spatial positions (the words “left” and “right”).

We predicted a larger influence of modality compatibility on mixing costs and switch costs with spatial-verbal stimuli compared to spatial-location stimuli. Since we attribute such effects of modality compatibility to crosstalk between the tasks, which can only arise in mixed-task blocks, we did not predict any particular impact of modality compatibility on single-task performance. However, we still included single-task blocks in the experiment to be able to calculate mixing costs. Like in previous studies (Stephan & Koch, 2010, 2011, 2015, 2016), we collapsed the data across both modality-compatible tasks and across both modality-incompatible tasks to equate any trivial processing differences between different stimulus modalities (visual vs. auditory) or response modalities (manual vs. vocal) by themselves (such as vocal responses being generally slower than manual responses), since the term modality compatibility refers specifically to the interaction of stimulus modality and response modality (see Stephan & Koch, 2015, 2016).

Schacherer and Hazeltine (2019; see also Maquestiaux, Ruthruff, Defer, & Ibrahime, 2018) suggested that compatible modality mappings could be maintained separately in a visual-spatial subsystem and an auditory-verbal subsystem (e.g. Baddeley, 1992, 2010); thus, when two tasks have to be maintained in working memory at the same time (Los, 1996), the load would still be lower for two compatible mappings than for two incompatible modality mappings. Hence, we expected that task confusion with incompatible modality mappings would affect both mixing costs and switch costs because between-task crosstalk should generally be larger in mixed-task blocks. However, switch trials should lead to particularly strong crosstalk-based interference because of the recent activation of the competing modality mapping.

### Method

#### Participants

24 subjects were tested^1^ (21 female, 23 right-handed; mean age = 22.08, SD = 2.858, age range = 19–31). All of them reported normal or corrected-to-normal vision and hearing. Participants gave their informed consent and were compensated (received 6 € or partial course credit) for participating in the study. Both experiments were conducted in accordance with the ethical principles of the Declaration of Helsinki.

#### Stimuli and apparatus

The experiment was programmed using version 1.83.03 of PsychoPy2 (Peirce et al., 2019) and ran on a Linux computer using a 15.4″ screen. Auditory spatial-location stimuli were generated in the software Audacity. Spatial-verbal auditory stimuli were recorded in a non-reflecting chamber.

**Fig. 1:**
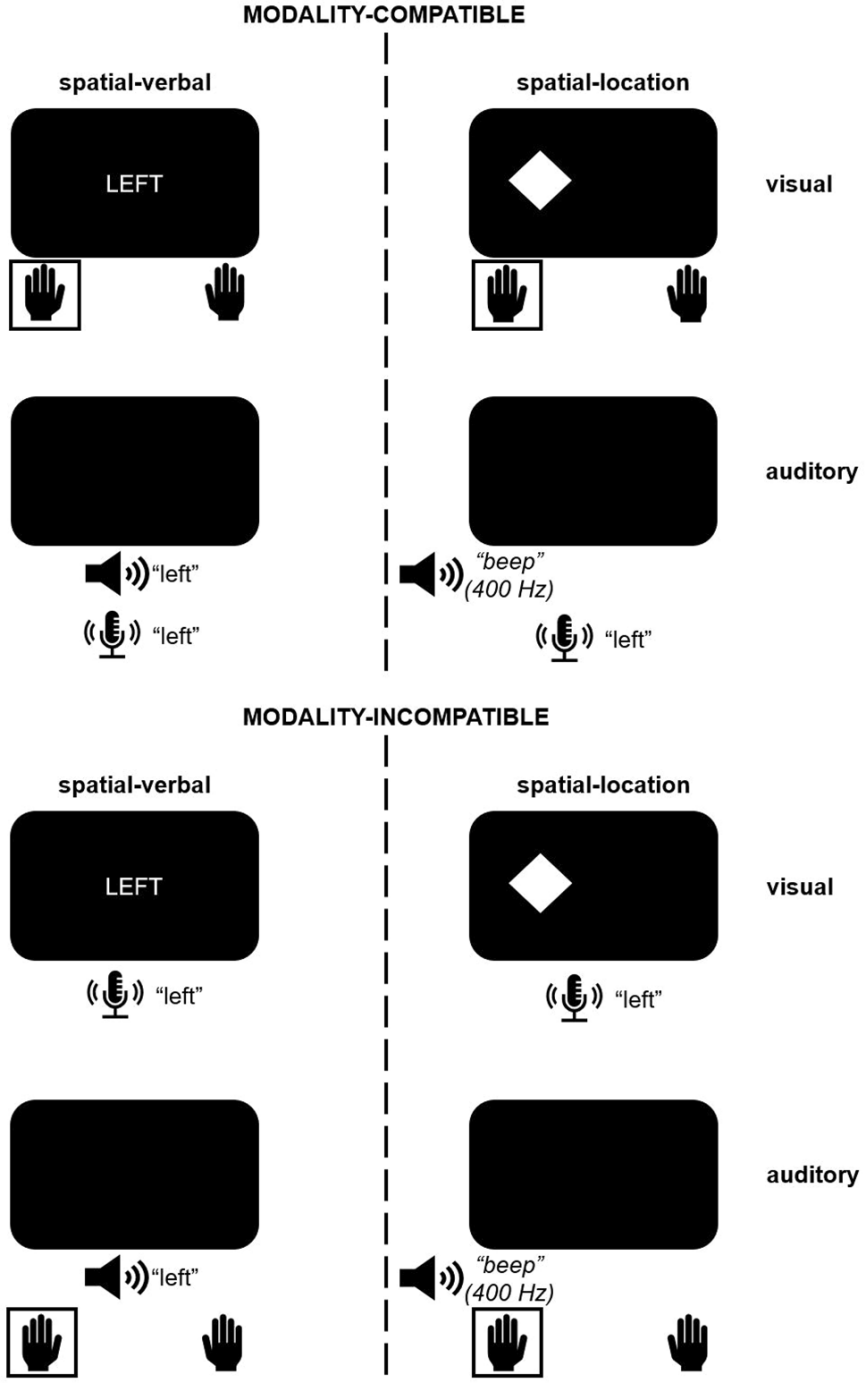
Overview of stimulus–response mappings in the modality-compatible and modality-incompatible condition for spatial-verbal and spatial-location processing codes (Experiment 1). Spatial-verbal stimuli were presented centrally/binaurally, spatial-location stimuli on one ear/one side of the screen. In Experiment 2, spatial-verbal processing codes were replaced with temporal-duration task demands, while the spatial-location task demands remained the same

Figure 1 provides an overview over all possible combinations of stimuli and responses. Visual spatial-location stimuli were white diamonds of 1.5 cm in width and height, presented on a black background, either 1.25 cm to the left or right of the centre of the screen. Auditory spatial-location stimuli were beep tones at 400 Hz presented via headphones on either the left or right ear. Visual spatial-verbal stimuli were the German words “LINKS” (left) and “RECHTS” (right) presented centrally in white capital letters and also 1.5 cm in height. Auditory spatial-verbal stimuli were the same German words in spoken form and presented binaurally. All stimuli lasted until a response occurred, or, in case of auditory spatial-verbal stimuli, for the duration of the spoken words, which was comparable since both words were monosyllabic. No visual fixation cross was presented to prevent priming of the visual modality. The decision to present stimuli until a response occurred (which was possible for all conditions except auditory spatial-verbal trials) was made to remain consistent with previous studies using the spatial-location paradigm (Fintor, Poljac, Stephan, & Koch, 2018; Fintor, Stephan, & Koch, 2018a, 2018b; Stephan & Koch, 2010, 2015, 2016). We did not see any reason to assume that a longer presentation duration for the visual spatial-verbal stimuli compared to the auditory spatial-verbal stimuli should have a specific effect on modality compatibility.

#### Procedure

Each trial began with the presentation of the target stimulus for a maximum duration of 1500 ms; stimulus presentation stopped when a response was detected. Depending on the instructed modality mapping (compatible vs. incompatible), a visual or auditory stimulus required a vocal or a manual response. Because modality compatibility was blocked, no explicit cues were required to instruct the response modality. The spatial stimulus–response (S–R) mapping was compatible for all subjects (that is, a left stimulus always required a left response, a right stimulus a right response). Manual responses were button presses with the left and right index finger; vocal responses were the German words “links” (= left) and “rechts” (= right) and recorded via a microphone, with both the microphone and the board featuring the buttons being connected to a response box. After a response had occurred, a response-stimulus interval (RSI) of 600 ms followed. Accuracy of vocal responses was coded by the experimenter during this interval; this allowed for error feedback to be presented after incorrect responses in either response modality. In case of an incorrect response (which included the case of a response on the correct spatial side but in the wrong modality), error feedback was presented bimodally—a red exclamation mark in the centre of the screen and a binaurally presented “boing” sound, both for 400 ms, after the standard RSI of 600 ms. Bimodal, nonverbal and centrally/binaurally presented error feedback was selected to ensure the error message would neither prime one modality (visual or auditory) nor one processing code (spatial-verbal or spatial-location) over the other. Error feedback was followed by a blank screen and silence on the headphones for 100 ms, lengthening the total RSI in case of an error to 1100 ms.

**Fig. 2:**
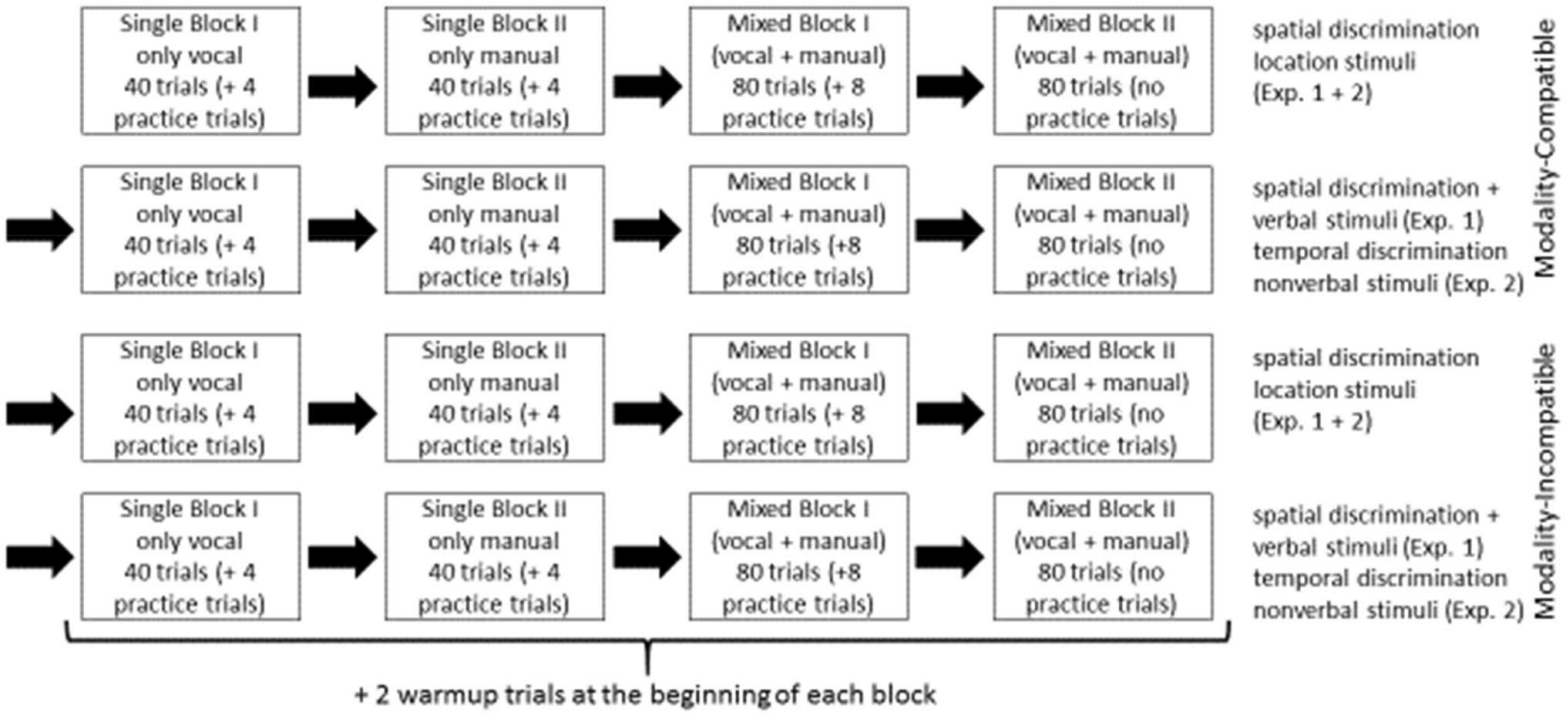
Example procedure of the whole experiment. Order of modality-compatibility conditions, processing code (spatial-location vs. spatial-verbal) in Experiment 1/task demand (spatial-location vs. temporal-duration) in Experiment 2, and response modalities in the single-task blocks was counterbalanced across all participants (Exp. 1 + 2 = Experiment 1 and Experiment 2)

The experiment was split in halves, a modality compatible and an incompatible condition (see Fig. 2). Within each condition, spatial-verbal and spatial-location stimuli were blocked, in counterbalanced order. Within each processing code condition, the block sequence was two single-task blocks of 40 trials each, one for each response modality (vocal and manual), followed by two mixed-task blocks of 80 trials each, featuring switches between the two previously introduced tasks. This means that within a given block, participants either switched between two compatible modality mappings (visual-manual and auditory–vocal), or between two incompatible modality mappings (visual–vocal and auditory-manual), responding to either only written and spoken words (spatial-verbal) in some blocks, or only to left and right diamond shapes and beep sounds (spatial-location) in other blocks. There were additional practice trials, 4 for each single-task block, 8 for the first of each two identical mixed-task blocks, as well as 2 warm-up trials after practice, at the beginning of the proper test phase of each block. The order of modality compatibility, processing code, and response modality (regarding which single-task came first) was counterbalanced across all participants. The overall duration of the experiment was about 45 min.

#### Design

The experiment had a within-subjects design with the independent variables processing code (spatial-verbal vs. spatial-location), modality compatibility (compatible vs. incompatible), and transition (repetitions in mixed-task blocks vs. single-task blocks for the mixing-cost contrast; switch vs. repetition in mixed-task blocks for the switch-cost contrast). The dependent variables were RT and error rates. All analyses were conducted at *α* = 0.05.

Note that task-switching costs in our study refer to switching between modality mappings, so that a switch in stimulus modality always also entailed a switch in response modality. Results were analysed averaged across both compatible modality mappings (visual-manual vs. auditory–vocal) vs. across both incompatible modality mappings (visual–vocal vs. auditory manual), so that the main effect of modality compatibility describes the difference in RT and error rates between the average of the two compatible modality mappings and the average of the two incompatible modality mappings. As such, our modality-compatibility contrast is independent of shifts in stimulus modality and response modality per se because both are strictly comparable when switching between modality incompatible modality mappings and when switching between modality compatible mappings.

### Results

The practice trials and the first two test trials of each block were excluded from the analysis, as well as all trials with RT outside ± 3*z* around the mean per participant and block, and/or RT below 50 ms (0.003% of the data); RT analysis excluded all error trials and trials following an error trial, and error analysis excluded trials succeeding an error trial.

For the single-task blocks, we ran a repeated-measures ANOVA with the variables modality compatibility (incompatible vs. compatible) and processing code (spatial-verbal vs. spatial-location). For the analysis of mixed-task blocks, we calculated two ANOVAs each for RT and error rates, involving the independent variables modality compatibility (incompatible vs. compatible), processing code (spatial-verbal vs. spatial-location), and transition ([mixed repeat vs. single task {which is repetition by definition} for the mixing-cost contrast]; [switch vs. repetition for the switch-cost contrast]). To follow up significant interactions, paired-sample *t*-tests were calculated. All analyses were conducted at *α* = 0.05.

#### Single-task analysis

**Fig. 3:**
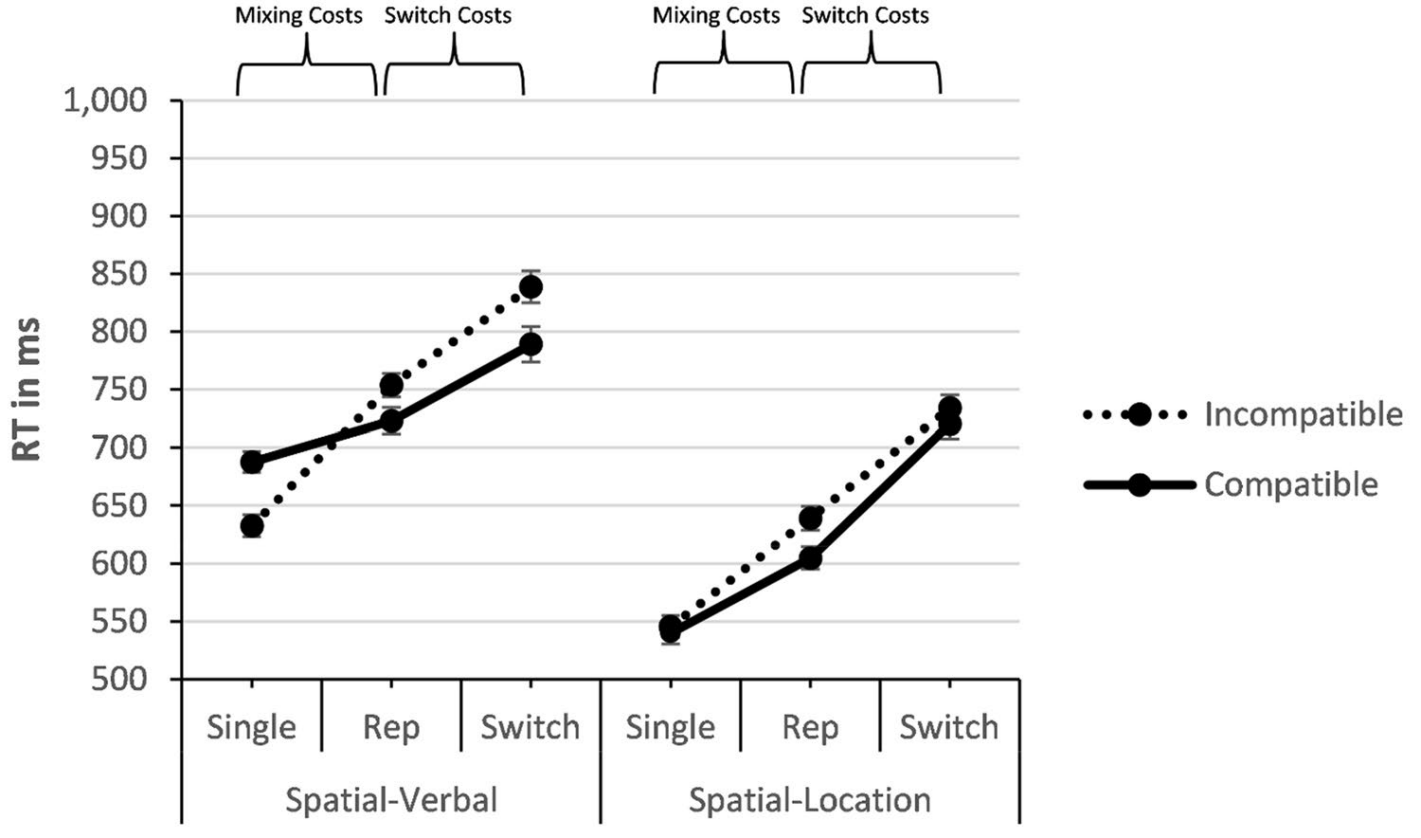
Mean response times (RTs) in ms in Experiment 1 in the mixing-cost contrast and the switch-cost contrast (*Rep * = Repetition). Error bars indicate the standard error of the mean

The ANOVA for single-task RT yielded a significant main effect of modality compatibility, *F*(1, 23) = 9.546, *p* = 0.005, *η*_*p*_^2^ = 0.293, showing higher RT on modality-compatible trials than incompatible trials (614 ms vs. 589 ms). Thus, any differences between modality-compatible mappings and modality-incompatible mappings in the task-switching analysis cannot be attributed to higher RT, due to greater single-task difficulty, for modality incompatible mappings (see Fig. 3). The effect of processing code was also significant, *F*(1, 23) = 524.833, *p* < 0.001, *η*_*p*_^2^ = 0.958, revealing slower responses for spatial-verbal stimuli than for spatial-location stimuli (660 ms vs. 543 ms). Finally, there was a significant interaction of modality compatibility and processing code, *F*(1, 23) = 26.256, *p* < 0.001, *η*_*p*_^2^ = 0.533, indicating that the modality-compatibility effect was larger for spatial-verbal than for spatial-location stimuli (− 56 ms vs. 5 ms).

The single-task analysis of error rates yielded a significant effect of modality compatibility, *F*(1, 23) = 13.776, *p* = 0.001, *η*_*p*_^2^ = 0.375, showing more errors for modality compatible than incompatible trials (2.6% vs. 1.5%) (see Table 1). There was also an effect of processing code, *F*(1, 23) = 19.693, *p* < 0.001, *η*_*p*_^2^ = 0.461, revealing higher error rates for spatial-verbal than for spatial-location stimuli (3.0% vs. 1.1%). Finally, modality compatibility interacted significantly with processing code, *F*(1, 23) = 15.221, *p* = 0.001, *η*_*p*_^2^ = 0.398, indicating a larger modality-compatibility effect for spatial-verbal stimuli than for spatial-location stimuli (− 2.3% vs. 0.1).

**Table 1:** Mean PE (percent errors) in Experiment 1

MC	Transition	Spatial-location		Spatial-verbal	
		Mean PE	SD	Mean PE	SD
Modality-compatible	Switch	5.4	4.7	6.2	5.6
	Repetition	2.6	2.3	3.9	4.0
	Single	1.1	1.3	4.1	2.8
Modality-incompatible	Switch	7.9	7.1	7.7	7.6
	Repetition	2.5	2.9	3.6	3.2
	Single	1.2	1.7	1.8	2.4

*MC* modality compatibility

Together, there was a performance cost for modality-compatible mappings only when using spatial-verbal stimuli, but this was not the case when using spatial-location stimuli, which is consistent with previous studies using such stimuli (e.g. Stephan & Koch, 2010). Note that single-task conditions did not produce any benefit for modality-compatible mappings (and even a disadvantage with spatial-verbal stimuli), but our predictions refer to conditions that include mapping switches.

#### Mixing-cost contrast

The mixing-cost analysis of the RTs yielded a significant main effect of mixing, *F*(1, 23) = 209.028, *p* < 0.001, *η*_*p*_^2^= 0.901, revealing higher RT in the repetition trials in mixed-task blocks compared to single-task blocks (680 ms vs. 601 ms). The main effect of modality compatibility was not significant (*F* < 1). Processing code yielded a significant main effect, *F*(1, 23) = 389.147, *p* < 0.001, *η*_*p*_^2^= 0.944, indicating slower responses for spatial-verbal than for spatial-location stimuli (699 ms vs. 582 ms).

The expected two-way interaction of modality compatibility and mixing was significant, *F*(1, 23) = 39.630, *p* < 0.001, *η*_*p*_^2^= 0.633, showing larger mixing costs for the modality-incompatible condition than for the modality-compatible condition (107 ms vs. 50 ms). The interaction of modality compatibility and processing code was significant, too, *F*(1, 23) = 12.504, *p* = 0.002, *η*_*p*_^*2*^= 0.352, implying a larger modality-compatibility effect for spatial-location than for spatial-verbal stimuli (20 ms vs. − 12 ms). The interaction of processing code and mixing was non-significant (*F* < 1). Notably, the predicted three-way interaction of modality compatibility, mixing, and processing code was significant as well, *F*(1, 23) = 15.167, *p* = 0.001, *η*_*p*_^2^= 0.397. The post hoc *t-*test confirmed that mixing costs were significantly larger in the modality-incompatible condition than in the modality-compatible condition with spatial-verbal processing codes, *t*(23) = 8.247, *p* < 0.001, *d* = 1.68 (121 ms vs. 36 ms), whereas this difference was still significant but just smaller with spatial-location processing codes, *t*(23) = 2.241, *p* = 0.035, *d* = 0.46 (94 ms vs. 65 ms).

The error analysis showed a significant effect of mixing, *F*(1, 23) = 6.914, *p* = 0.015, *η*_*p*_^2^= 0.231, revealing more errors in repetition trials in mixed-task blocks than in single-task blocks (3.2% vs. 2.1%). There was a non-significant trend towards an effect of modality compatibility, *F*(1, 23) = 3.096, *p* = 0.092, *η*_*p*_^2^= 0.119, suggesting numerically higher error rates in the modality-compatible than in the incompatible condition (2.9% vs. 2.3%). There was also a significant effect of processing code, *F*(1, 23) = 20.983, *p* < 0.001, *η*_*p*_^2^= 0.477, with more errors for spatial-verbal than for spatial-location stimuli (3.4% vs. 1.9%).

The two-way interaction between modality compatibility and mixing was not significant, *F*(1, 23) = 1.684, *p* = 0.207, *η*_*p*_^2^= 0.068; however, mixing costs were still numerically larger in the modality-incompatible condition (1.6%) than in the modality-compatible condition (0.6%). The interaction of processing code and mixing was non-significant, *F*(1, 23) = 2.496, *p *= 0.128, *η*_*p*_^2^= 0.098. There was a non-significant trend for an interaction between modality compatibility and processing code, *F*(1, 23) = 3.643, *p* = 0.069, *η*_*p*_^2^ = 0.137, but importantly, like in the RT data, modality compatibility, mixing, and processing code entered a significant three-way interaction, *F*(1, 23) = 5.555, *p* = 0.027, *η*_*p*_^2^= 0.195. The post hoc *t*-test confirmed that spatial-verbal processing codes led to larger mixing costs in the modality-incompatible condition compared to the modality-compatible condition, *t*(23) = 2.069, *p* = 0.050, *d* = 0.42 (1.8% vs. − 0.3%), whereas spatial-location processing codes showed no significant difference in mixing cost between modality-incompatible and modality-compatible conditions, *t*(23) = 0.293, *p* = 0.772, *d* = 0.06 (1.3% vs. 1.5%).

#### Switch-cost contrast

The RT task-switching analysis in the switch-cost contrast yielded a significant effect of switching, *F*(1, 23) = 200.725, *p* < 0.001, *η*_*p*_^2^= 0.897, revealing higher RT on switch trials than on repetition trials (771 ms vs. 680 ms). There was also an effect of modality compatibility, *F*(1, 23) = 12.218, *p* < 0.002, *η*_*p*_^2^= 0.347, with higher RT for the modality-incompatible condition than for the modality-compatible condition (741 ms vs. 709 ms). Finally, there was a main effect of processing code, *F*(1, 23) = 246.681, *p* < 0.001, *η*_*p*_^2^= 0.915, showing higher RT for spatial-verbal processing codes than for spatial-location processing codes (776 ms vs. 675 ms).

The interaction of modality compatibility and switching was not significant, *F*(1, 23) = 0.004, *p* = 0.953, *η*_*p*_^*2*^= 0.000, but switching interacted significantly with processing code, *F*(1, 23) = 12.310, *p* = 0.002, *η*_*p*_^2^= 0.349, showing larger switch costs with spatial-location codes than with spatial-verbal codes (105 ms vs. 76 ms). The interaction of processing code and modality compatibility was non-significant, *F*(1, 23) = 1.995, *p* = 0.171, *η*_*p*_^2^= 0.080, but the three-way interaction of modality compatibility, switching, and processing code was significant, *F*(1, 23) = 7.074, *p* = 0.014, *η*_*p*_^2^= 0.235. The post hoc *t*-test showed (non-significantly) larger switch costs in the modality-incompatible condition relative to the modality-compatible condition (85 ms vs. 66 ms) for spatial-verbal processing codes, *t*(23) = 0.999, *p* = 0.328, *d* = 0.20, whereas for spatial-location processing codes there was an unexpected non-significant trend in the opposite direction, *t*(23) = 2.001, *p* = 0.057, *d* = 0.41 (116 ms modality-compatible vs. 95 ms modality-incompatible). Note that the error rates show increased switch costs in the modality-incompatible condition for the spatial-location processing codes, implying a specific speed-accuracy trade-off (see below).

The analysis of error rates in the switch-cost contrast showed that switching led to a significant main effect, *F*(1, 23) = 23.260, *p* < 0.001, *η*_*p*_^2^= 0.503, revealing more errors on switch trials than on repetition trials (6.8% vs. 3.2%). There was an effect of processing code, *F*(1, 23) = 4.455, *p* = 0.046, *η*_*p*_^2^= 0.162, revealing higher error rates for spatial-verbal than for spatial-location processing codes (5.3% vs. 4.6%). The interaction between modality compatibility and switching was significant, *F*(1, 23) = 5.795, *p* = 0.024, *η*_*p*_^2^= 0.201, revealing larger switch costs in the modality-incompatible condition compared to the modality-compatible condition (4.7% vs. 2.6%). However, the three-way interaction together with processing code was not significant, *F*(1, 23) = 0.270, *p* = 0.608, *η*_*p*_^2^= 0.012, suggesting no difference in the effect of modality compatibility on switch costs between spatial-verbal and spatial-location stimuli. All other effects were non-significant (*F*s < 2).

Please note that the error data show generally larger switch costs for modality-incompatible conditions, regardless of whether processing codes were spatial-verbal or spatial-location. Notably, the opposing RT trend with spatial-location stimuli was thus clearly not supported by the error rates, Hence, the data of the switch cost contrast are ambiguous with spatial-location stimuli, suggesting a speed-accuracy trade-off that is not easily explainable, whereas the data show consistently increased switch costs with modality-incompatible mappings when using spatial-verbal stimuli. For the full overview of the data in each component task, please see Table 2.

**Table 2:** Mean RT (in ms) and PE in Experiment 1 for each component task (= with the factor modality compatibility split up into stimulus modality and response modality)

Stimulus modality	Response modality	Transition	Spatial-location		Spatial-verbal	
			Mean	SD	Mean	SD
RT						
Visual	Manual	Switch	483.75	59.463	559.00	90.833
		Repetition	380.13	49.519	481.42	57.194
		Single	314.75	30.543	425.96	41.215
Auditory	Vocal	Switch	961.08	87.358	1021.33	78.692
		Repetition	867.42	63.859	990.75	73.844
		Single	784.17	80.180	944.96	76.498
Visual	Vocal	Switch	821.63	87.678	817.63	67.395
		Repetition	701.67	60.507	729.63	59.504
		Single	623.38	58.267	647.17	56.451
Auditory	Manual	Switch	651.13	64.335	860.17	85.798
		Repetition	585.96	75.954	776.42	59.407
		Single	469.25	50.989	617.33	51.270
PE						
Visual	Manual	Switch	3.7171	5.16421	7.7502	4.70865
		Repetition	0.9294	1.62830	4.5419	5.40590
		Single	0.4090	0.93438	6.8457	4.03735
Auditory	Vocal	Switch	7.0014	6.13946	4.6185	9.02277
		Repetition	4.5722	3.92233	3.2810	4.30273
		Single	1.7678	2.63794	1.3849	2.55705
Visual	Vocal	Switch	11.6783	8.16878	7.7129	8.09657
		Repetition	3.4612	3.51095	3.8374	4.58211
		Single	0.5437	1.70025	0.9607	2.28423
Auditory	Manual	Switch	3.9118	6.10283	7.4676	8.20968
		Repetition	1.7895	3.52031	3.6602	4.18811
		Single	1.8706	2.50593	2.6972	3.53486

### Discussion

In Experiment 1, we tested the prediction that modality-compatibility effects in task switching should be larger with spatial-verbal stimuli because these should strengthen particularly the vocal-auditory modality mapping. We found a consistent influence of spatial-verbal stimuli in terms of a larger modality-compatibility effect on mixing costs compared to spatial-location stimuli. We also found increased switch costs with modality incompatibility with spatial-verbal stimuli (even though the trend was non-significant in RT), but for spatial-location stimuli we found it only for error rates, whereas this effect was even non-significantly reversed in RT, hinting at a specific speed-accuracy trade-off. Note that the effect of increased RT switch costs in modality-incompatible conditions has been replicated several times with spatial-location stimuli (Fintor, Stephan et al., 2018a; Stephan & Koch, 2010, 2011, 2015, 2016), so that its absence in the present experiment should not be overemphasized.

Note also that the single-task blocks showed even worse performance in modality-compatible conditions, but this was confined to spatial-verbal conditions. While we have no explanation for this effect, methodologically it rules out that more “difficult” tasks also lead to larger costs in multitasking (Stephan & Koch, 2011) because the data pattern is the opposite.

Using a dual-task setting, Göthe et al. (2016) had performed a modality-compatibility study with spatial-verbal and spatial-location stimuli in a between-subjects design with bimodal stimulation. They found higher dual-task costs not only for modality-incompatible mappings, but also for location-vocal and verbal-manual mappings of processing code and response, compared to the mappings spatial-manual and verbal-vocal. Notably, the highest costs were observed in the group that faced both an incompatible modality mapping and a location-vocal + verbal-manual feature mapping. In our task-switching setup, all factors were varied within subjects. Note also that none of the mappings in the study by Göthe et al. (2016) met the narrow definition of ideomotor compatibility (Greenwald, 1972), whereas our setup included one ideomotor-compatible condition: the auditory-verbal condition, that is, hearing the word “left” or “right” and responding vocally by saying the same word.

Taken together, the data suggest that spatial-verbal stimuli create greater between-task crosstalk than spatial-location stimuli when two tasks with modality-incompatible stimulus–response mappings have to be maintained in working memory at the same time. Note though that the spatial-verbal and the spatial-location task we used in Experiment 1 were similar to each other because both types of stimuli contained a spatial meaning (left vs. right).

## Experiment 2

In Experiment 2 we compared performance with the spatial-discrimination task with that in a genuinely different type of discrimination, namely a temporal-discrimination task. By employing a task that should be more appropriate for the auditory modality in a multitasking setting (Freides, 1974; Lukas et al., 2014; Talsma, Senkowski, Soto-Faraco, & Woldorff, 2010; Welch & Warren, 1980), once more, like in Experiment 1, we expected the auditory–vocal coupling to be strengthened: The results pattern found for spatial-verbal processing codes and temporal-duration task demands should be comparable, since segmenting speech also requires high temporal resolution (e.g. Bell-Berti & Harris, 1981; Smith, 1978). The likelihood of the anticipation of the effect of a vocal response priming the processing of auditory input should therefore increase, since the auditory modality is the relatively more appropriate one for temporal processing in a multitasking setting to begin with. If such a prime for auditory input occurs when the actual stimulus is visual, the primed task would be the wrong one. Thus we again predicted that mixing costs and switch costs should be larger with modality-incompatible mappings, and this should be even more pronounced with a temporal-duration task than with a spatial-location task.

### Method

#### Participants

24 new participants who had not taken part in Experiment 1 were tested (18 female, 23 right-handed; mean age = 22.26, SD = 2.490, age span = 19–29). All of them reported normal or corrected-to-normal eyesight and hearing. Each of them gave their informed consent and received 6 € or partial course credit for their participation.

#### Stimuli and apparatus

Stimuli for the spatial-discrimination task were the same spatial-location stimuli as in Experiment 1. For the temporal-discrimination task, as visual stimuli we used the same diamonds as for the spatial-discrimination task, but presented centrally for either 100 ms (short) or 500 ms (long). The auditory equivalent was the same beep tone as in the spatial-discrimination task (400 Hz), presented binaurally for the respective durations (with parameters borrowed from Lukas et al., 2014). Like in Experiment 1, we used a constant spatially-compatible S–R mapping for all participants, that is, in the spatial-location task, a left stimulus required a left response, a right stimulus a right response. For the temporal-duration task, we argue that left is spatially more compatible with short and right is spatially more compatible with long stimuli (e.g. Walsh, 2003).

#### Procedure

Trials and the overall experiment followed the same structure as Experiment 1, with the spatial-location condition remaining exactly as in Experiment 1, but with a temporal-duration task replacing the condition with spatial-verbal processing codes. As in Experiment 1, in both tasks, vocal responses were the words “links” (German for “left”) and “rechts” (German for “right”), and manual responses were left and right button presses.

Note that spatial-location stimuli were presented until a response occurred, but the temporal-duration stimuli were presented for constant durations (100 ms vs. 500 ms). Responses for both task demands were possible starting from stimulus onset, so in case a participant was able to identify a long-duration stimulus before 500 ms had elapsed, the stimulus would continue to be presented into the RSI. Once the stimulus had been presented for its designated duration, the remainder of the RSI consisted of silence and a blank screen.

As such, it needs to be considered that the earliest point at which a judgement of the temporal duration of the stimulus could be made was after 100 ms had elapsed. Even though subjects were told to respond as quickly and accurately as possible, we cannot rule out that, in case of a long-duration stimulus, some participants may have waited for the entire 500 ms before judging it as “long”. This means that overall RT for long stimuli was by design higher than for short stimuli, so that overall RT for temporal-duration tasks was also higher than overall RT for spatial-location tasks. Consequently, interpretations of main effects of task demand (temporal-discrimination vs. spatial-location-discrimination) are not meaningful, but interactions of task demand with modality compatibility and transition can be interpreted.

#### Design

The independent within-subjects variables were task demand (temporal-discrimination task vs. spatial-location-discrimination task), modality compatibility (compatible vs. incompatible), and transition (repetitions in mixed-task blocks vs. single-task blocks for the mixing-cost contrast; switch vs. repetition trials in the mixed-task blocks for the switch-cost contrast). The dependent variables were RT and error rates.

### Results

Data analysis proceeded as in Experiment 1. Trials excluded as RT outliers amounted to 0.002%.

#### Single-task analysis

**Fig. 4:**
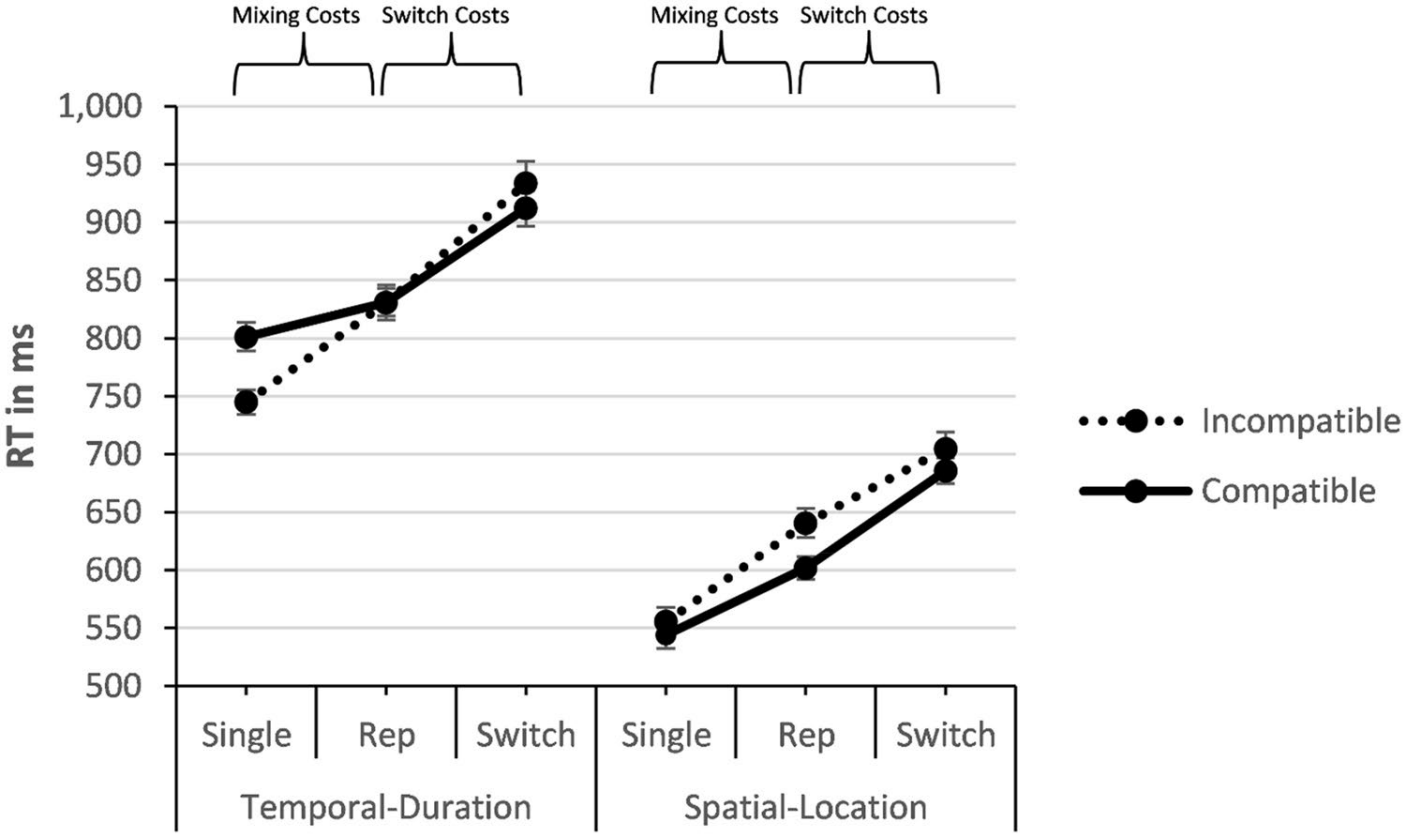
Mean RTs in ms in Experiment 2 in the mixing-cost contrast and the switch-cost contrast. Error bars indicate the standard error of the mean

The ANOVA on RT (see Fig. 4) yielded a significant effect of modality compatibility, *F*(1, 23) = 12.683, *p* = 0.002, *η*_*p*_^2^ = 0.355, revealing slower responses in the modality-compatible condition than in the modality-incompatible condition (673 ms vs. 650 ms). There was also an effect of task demand, *F*(1, 23) = 728.613, *p* < 0.001, *η*_*p*_^2^ = 0.969, indicating higher RT for temporal-duration task demands than for spatial-location task demands (773 ms vs. 550 ms). Finally, there was a significant interaction of modality compatibility and task demand, *F*(1, 23) = 18.975, *p* < 0.001, *η*_*p*_^2^ = 0.452, indicating a larger and reversed modality-compatibility effect with temporal-duration task demands compared to spatial-location task demands (− 56 ms vs. 11 ms).

The error-rate analysis (see Table 3) showed a significant effect of modality compatibility, *F*(1, 23) = 7.474, *p* = 0.012, *η*_*p*_^2^ = 0.245, revealing more errors on modality compatible than incompatible conditions (3.1% vs. 1.9%). There was also an effect of task demand, *F*(1, 23) = 75.366, *p* < 0.001, *η*_*p*_^2^ = 0.766, pointing at higher error rates with temporal than with spatial-location discrimination (4.1% vs. 0.9%). Finally, there was a non-significant trend towards an interaction of modality compatibility and task demand, *F*(1, 23) = 3.755, *p* = 0.065, *η*_*p*_^2^ = 0.140, suggesting a numerically larger modality-compatibility effect for temporal-duration task demands than for spatial-location task demands (− 1.9% vs. − 0.4%).

**Table 3:** Mean PE in Experiment 2

MC	Transition	Spatial-location		Temporal-duration	
		Mean PE	SD	Mean PE	SD
Modality-compatible	Switch	3.0	3.1	7.2	4.9
	Repetition	2.3	2.6	7.1	4.1
	Single	1.1	1.4	5.1	3.3
Modality-incompatible	Switch	5.9	4.6	8.6	5.5
	Repetition	2.6	2.6	3.8	2.9
	Single	0.7	1.1	3.2	2.6

#### Mixing-cost contrast

The RT analysis yielded a main effect of mixing, *F*(1, 23) = 106.257, *p* < 0.001, *η*_*p*_^2^ = 0.822, showing larger RT for repetitions in mixed-task blocks compared to single-task blocks (726 ms vs. 661 ms). There was also a main effect of task demand, *F*(1, 23) = 1103.394, *p* < 0.001, *η*_*p*_^2^ = 0.980, revealing higher RT for temporal-duration task demands than for spatial-location task demands (802 ms vs. 585 ms).

There was an interaction of modality compatibility and mixing, *F*(1, 23) = 23.109, *p* < 0.001, *η*_*p*_^2^ = 0.501, confirming larger mixing costs in the modality-incompatible condition compared to the modality-compatible condition (86 ms vs. 43 ms). Modality compatibility and task demand also interacted significantly, *F*(1, 23) = 19.964, *p* < 0.001, *η*_*p*_^2^ = 0.465, implying a reversed overall influence of modality compatibility in the temporal-duration task compared to an influence of modality compatibility in the expected direction with the spatial-location task (-28 ms temporal-duration vs. 25 ms spatial-location).

Notably, for the predicted three-way interaction of modality compatibility, task demand, and mixing we found a non-significant trend, *F*(1, 23) = 3.709, *p* = 0.067, *η*_*p*_^2^ = 0.139. Despite this non-significant result, we calculated a follow-up test to determine whether at least the direction of this numerical trend was consistent with our hypothesis, since we had indeed found this significant three-way interaction in the mixing-cost contrast of both RT and error rates in Experiment 1. These post hoc *t-*tests suggested that the modality-compatibility effect on mixing costs tended to be larger for the temporal-duration task demand, *t*(23) = 5.313, *p* < 0.001, *d* = 1.08 (86 ms mixing costs for the modality-incompatible condition vs. 30 ms mixing costs for the modality-compatible condition) than for the spatial-location task demand, *t*(23) = 2.221, *p* = 0.037, *d* = 0.45 (85 ms modality-incompatible vs. 58 ms modality-compatible). All other effects were non-significant (*F*s < 2.2, *p*s > 0.1).

The error analysis yielded an effect of mixing, *F*(1, 23) = 21.298, *p* < 0.001, *η*_*p*_^2^ = 0.481, indicating more errors for repetitions in mixed-task blocks compared to single-task blocks (4.0% vs. 2.5%). Modality compatibility yielded a significant effect as well, *F*(1, 23) = 8.135, *p* = 0.009, *η*_*p*_^2^ = 0.261, revealing more errors in the modality-compatible compared to the modality-incompatible condition (3.9% vs. 2.6%). There was also an effect of task demand, *F*(1, 23) = 95.006, *p* < 0.001, *η*_*p*_^2^ = 0.805, showcasing higher error rates with temporal-duration task demands than with spatial-location task demands (4.8% vs. 1.7%). Furthermore, there was a significant interaction between modality compatibility and task demand, *F*(1, 23) = 9.078, *p* = 0.006, *η*_*p*_^2^ = 0.283, suggesting that the (reversed) modality-compatibility effect was present with temporal-duration but not with spatial-location task demands (− 2.6% vs. 0.0%).

The three-way interaction of modality compatibility, task demand, and mixing again showed a non-significant trend, *F*(1, 23) = 3.713, *p* = 0.066, *η*_*p*_^2^ = 0.139. Again, a post hoc *t*-test was calculated to determine the direction of this numerical trend; this test however showed that the effect of modality compatibility on mixing costs was neither significant for temporal-duration task demands, *t*(23) = 1.347, *p* = 0.191, *d* = 0.27 (0.7% modality-incompatible vs. 2.0% modality-compatible), nor for spatial-location task demands, *t*(23) = 1.064, *p* = 0.299, *d* = 0.22 (1.9% vs. 1.3%). All other effects were non-significant, including the interaction of modality compatibility and mixing (*F*s < 1).

#### Switch-cost contrast

The RT analysis revealed an effect of switching, *F*(1, 23) = 164.778, *p* < 0.001, *η*_*p*_^2^ = 0.878, with higher RT on switches than on repetitions (809 ms vs. 726 ms). There was also an effect of modality compatibility, *F*(1, 23) = 6.065, *p* = 0.022, *η*_*p*_^2^ = 0.209, showing slower responses for the modality-incompatible condition compared to the modality-compatible condition (777 ms vs. 758 ms). Finally, task demand revealed a significant effect as well, *F*(1, 23) = 1095.559, *p* < 0.001, *η*_*p*_^2^ = 0.979, showing higher RT for temporal-duration task demands than for spatial-location task demands (877 ms vs. 658 ms).

We found a significant interaction between task demand and switching, *F*(1, 23) = 5.727, *p* = 0.025, *η*_*p*_^2^ = 0.199, revealing larger switch costs for temporal-duration task demands than for spatial-location task demands (92 ms vs. 74 ms). Note that modality compatibility and switching did not show a significant interaction, *F*(1, 23) = 0.011, *p* = 0.916, *η*_*p*_^2^ = 0.000, but the predicted three-way interaction of modality compatibility, task demand, and switching was significant, *F*(1, 23) = 11.122, *p* < 0.003, *η*_*p*_^2^ = 0.326. The post hoc *t*-test showed that for the temporal-duration task demand, switch costs were numerically larger in the modality-incompatible condition compared to the modality-compatible condition, *t*(23) = 1.538, *p* = 0.138, *d* = 0.31 (103 ms vs. 81 ms), but for spatial-location, switch costs were actually larger in the modality-compatible condition than in the modality-incompatible condition, *t*(23) = 2.593, *p* = 0.016, *d* = 0.53 (84 ms vs. 64 ms). The remaining interaction of modality compatibility and task demand was non-significant (*p* > 0.10).

The error analysis in the switch-cost contrast demonstrated a significant effect of switching, *F*(1, 23) = 15.521, *p* = 0.001, *η*_*p*_^2^ = 0.403, showing more errors on switch trials than on repetition trials (6.2% vs. 4.0%). There was a significant effect of task demand, too, *F*(1, 23) = 85.443, *p* < 0.001, *η*_*p*_^2^ = 0.788, revealing more errors for temporal-duration task demands than for spatial-location task demands (6.7% vs. 3.5%).

Unlike in the RT data, there was a significant interaction of modality compatibility and switching, *F*(1, 23) = 11.022, *p* = 0.003, *η*_*p*_^2^ = 0.324, confirming larger switch costs in the modality-incompatible condition compared to the compatible condition (4.1% vs. 0.4%). The interaction of modality compatibility and task demand was also significant, *F*(1, 23) = 7.314, *p* = 0.013, *η*_*p*_^2^ = 0.241, implying a stronger general modality-compatibility effect with spatial-location task demands than with temporal-duration task demands, for which the modality-compatibility effect was reversed (1.7% vs. − 0.9%). Yet, the three-way interaction of modality compatibility, task demand, and switching was not significant, *F*(1, 23) = 2.292, *p* = 0.144, *η*_*p*_^2^ = 0.091, suggesting no difference in the size of the modality-compatibility effect on switch costs between temporal-duration task demands and spatial-location task demands. All other effects were non-significant (*F*s < 1).

Again, like in Experiment 1, the data of Experiment 2 showed a reasonably clear picture in the mixing cost contrast, but in the switch cost contrast the data of the spatial-location task shows an unclear trade-off, with larger error switch costs in the modality-incompatible condition, which was expected, but unexpectedly smaller RT switch costs in the modality-incompatible condition. Hence, the influence of modality compatibility on switch costs with spatial-location task demands are again difficult to interpret.^2^ For the full overview of the data in each component task, please see Table 4.

**Table 4:** Mean RT (in ms) and PE in Experiment 2 for each component task (= with the factor modality compatibility split up into stimulus modality and response modality)

Stimulus modality	Response modality	Transition	Spatial-location		Temporal-duration	
			Mean	SD	Mean	SD
RT						
Visual	Manual	Switch	439.54	43.738	711.96	82.770
		Repetition	366.50	41.308	656.46	60.433
		Single	303.67	26.249	597.50	54.375
Auditory	Vocal	Switch	931.58	87.036	1112.46	75.790
		Repetition	873.13	76.978	1058.08	83.114
		Single	805.54	97.212	1006.13	85.068
Visual	Vocal	Switch	764.04	86.364	985.50	98.714
		Repetition	693.54	66.753	915.54	87.832
		Single	626.79	54.194	856.79	67.834
Auditory	Manual	Switch	647.13	80.539	882.33	110.135
		Repetition	594.63	74.174	759.50	74.035
		Single	486.04	89.453	639.08	57.989
PE						
Visual	Manual	Switch	2.3107	3.26491	9.7120	7.36968
		Repetition	1.1738	3.56109	6.9496	5.45941
		Single	0.7376	1.75193	7.6382	6.57806
Auditory	Vocal	Switch	3.6720	4.48619	4.3236	4.04629
		Repetition	3.6480	4.13481	7.3511	6.41058
		Single	1.4554	1.94290	2.9853	3.40751
Visual	Vocal	Switch	9.1799	7.40682	8.2989	7.61209
		Repetition	3.8544	4.07643	4.4368	5.23055
		Single	0.2360	0.80804	2.6429	6.10841
Auditory	Manual	Switch	2.4245	2.88267	8.8043	5.23203
		Repetition	1.5701	2.15064	3.3561	2.68115
		Single	1.1489	1.82094	4.0485	3.51741

### Discussion

In Experiment 2, we found an influence of temporal-duration task demands, in form of a trend for a stronger effect of modality compatibility on mixing costs compared to spatial-location task demands. However, the data suggested a numerically larger influence of modality compatibility on mixing costs with the temporal-duration task only for RT. Again, similarly to Experiment 1, for spatial-location task demands the switch cost contrast showed an unclear speed-accuracy trade-off. The predicted interactions of modality compatibility and transition revealed a modality-compatibility effect on mixing costs for RT, and a modality-compatibility effect on switch costs for errors.

## Supplemental analyses

To assess to what extent the auditory–vocal coupling was strengthened by spatial-verbal processing codes and temporal-duration task demands compared to spatial-location processing codes/task demands, we considered performance with the individual modality-incompatible mappings. Therefore, for the auditory-manual mapping and the visual–vocal mapping separately, we ran 2 × 2 ANOVAs with the within-subjects variables transition (mixed vs. single in the mixing-cost contrast and switch vs. repetition in the switch-cost contrast) and processing code (spatial-verbal vs. spatial-location) for Experiment 1 or task demand (temporal-duration vs. spatial location) for Experiment 2. For the sake of brevity, we only report the relevant interactions of mixing/switching and processing code/task demand.

### Auditory-manual mapping

For Experiment 1, the interaction of mixing and processing code was significant for RT, *F*(1, 23) = 6.708, *p* = 0.016, *η*_*p*_^2^ = 0.226, revealing larger mixing costs with spatial-verbal codes compared to spatial-location codes (159 ms vs. 117 ms); in error rates, the interaction was not significant (*p* = 0.263), but the numerical trend pointed into the same direction (1.0% spatial-verbal vs. − 0.1% spatial-location). The interaction of switching and processing code was neither significant for RT, *F*(1, 23) = 1.845, *p* = 0.188, *η*_*p*_^2^ = 0.074, nor for errors, *F*(1, 23) = 1.230, *p* = 0.279, *η*_*p*_^2^ = 0.051, but there were numerically larger switch costs with spatial-verbal processing codes than with spatial-location processing codes (84 ms vs. 65 ms for RT and 3.8% vs. 2.1% for errors).

For Experiment 2, the interaction of mixing and task demand was neither significant for RT, *F*(1, 23) = 0.746, *p* = 0.397, *η*_*p*_^2^ = 0.031, nor for errors, *F*(1, 23) = 1.905, *p* = 0.181, *η*_*p*_^2^ = 0.076. The interaction of switching and task demand however was significant in both RT, *F*(1, 23) = 40.251, *p* < 0.001, *η*_*p*_^2^ = 0.636, and error rates, *F*(1, 23) = 15.091, *p* = 0.001, *η*_*p*_^2^ = 0.396, with larger switching costs for the temporal-duration task demand than for the spatial-location task demand (122 ms vs. 52 ms for RT and 5.4% vs. 0.8% for errors).

### Visual–vocal mapping

For Experiment 1, the interaction of mixing and processing code was neither significant for RT, *F*(1, 23) = 0.147, *p* = 0.705, *η*_*p*_^2^ = 0.006, nor for errors, *F*(1, 23) = 0.001, *p* = 0.970, *η*_*p*_^2^ = 0.000. Switching and processing code, however, revealed a non-significant interaction trend in RT, *F*(1, 23) = 3.836, *p* = 0.062, *η*_*p*_^2^ = 0.143 (120 ms spatial-location vs. 88 ms spatial-verbal), and a significant interaction in error rates, *F*(1, 23) = 6.416, *p* = 0.019, *η*_*p*_^2^ = 0.218 (8.2% spatial-location vs. 3.9% spatial-verbal), demonstrating consistently higher switch costs for spatial-location codes than for spatial-verbal codes.

For Experiment 2, mixing and task demand did not interact significantly, neither for RT, *F*(1, 23) = 0.294, *p* = 0.593, *η*_*p*_^2^ = 0.013, nor for error rates, *F*(1, 23) = 1.438, *p* = 0.243, *η*_*p*_^2^ = 0.059. Switching and task demand neither showed a significant interaction for RT, *F*(1, 23) = 0.003, *p* = 0.960, *η*_*p*_^2^ = 0.000, nor for errors, *F*(1, 23) = 0.868, *p* = 0.361, *η*_*p*_^2^ = 0.036.

Taken together, these results reveal that mixing costs and switch costs with the modality-incompatible auditory-manual mapping were indeed negatively affected by both spatial-verbal processing codes and temporal-duration task demands compared to spatial-location processing codes/task demands. Specifically, with the auditory-manual mapping spatial-verbal processing codes predominantly affected mixing costs, whereas temporal-duration task demands mainly influenced switch costs. Meanwhile, mixing costs and switch costs with the competing visual–vocal mapping either did not differ based on processing code/task demand, or they were enlarged with the spatial-location processing code/task demand compared to its respective counterpart. This suggests that the increased influence of modality compatibility on mixing costs (and, to a lesser extent, switch costs) which we found in the main analysis with both spatial-verbal processing codes and temporal-duration task demands can be attributed mainly to their effect on the auditory-manual mapping, probably because auditory stimuli are particularly strongly coupled with vocal responses.

We can only speculate why the auditory-manual mapping was only affected in terms of mixing costs by the spatial-verbal processing codes and only in terms of switch costs by the temporal-duration task demands. The absence of an effect of temporal-duration task demands on mixing costs for the auditory-manual mapping might be explained by the smaller similarity between the auditory temporal-duration stimulus and the auditory effect of the response, which was still spatial-verbal (the spoken word “left” or “right”)—compared to Experiment 1, where the effect of the vocal response and the auditory stimulus were ideomotor-compatible. This smaller similarity in Experiment 2 might have led to less between-task crosstalk.

## General discussion

In two experiments, we examined modality compatibility in task switching using visual and auditory stimuli as well as manual and vocal responses. Previous studies demonstrated increased mixing costs and switch costs with incompatible modality mappings compared to compatible modality mappings. In the present study we investigated whether spatial-verbal processing codes (Experiment 1) and/or temporal-discrimination task demands (Experiment 2) would increase the strength of the auditory–vocal coupling and thereby the influence of modality compatibility on mixing and switch costs.

### Summary of main findings

We found a consistently larger modality-compatibility effect on mixing costs for both spatial-verbal codes compared to spatial-location codes (Experiment 1) and for temporal-discrimination task demands compared to spatial-location task demands (Experiment 2). The findings with respect to switch costs are less consistent in the spatial-discrimination paradigm because of an unexpected speed-accuracy trade-off, but for both spatial-verbal processing codes and temporal-duration tasks switch costs were always at least numerically larger in the modality-incompatible condition than in the modality-compatible condition.

The analysis of the individual modality mappings supported our account that, compared to spatial-location discrimination tasks (i.e. requiring spatial-location codes), both spatial-verbal codes and temporal-duration task demands indeed strengthened the auditory–vocal coupling. This is suggested by the analysis of the respective counterparts to those couplings, i.e. the individual modality-incompatible mappings: The auditory-manual mapping showed larger mixing costs with spatial-verbal processing codes and larger switch costs with temporal-duration task demands, as well as numerical trends into the same direction for larger switch costs with spatial-verbal processing codes in Experiment 1 and larger mixing costs with temporal-duration task demands in Experiment 2. Meanwhile, the overall impact of processing codes/task demands on the visual-manual coupling was much less pronounced when assessed in the modality-incompatible counterpart, that is, the visual–vocal mapping.

### The role of processing codes in mixing costs

Embedding our findings on mixing costs into the literature, our results are in line with Schacherer and Hazeltine (2019), who also found larger mixing costs with modality-incompatible mappings than with modality-compatible mappings. Our study suggests, more specifically, that both spatial-verbal processing codes and temporal-discrimination task demands increase the impact of modality compatibility on mixing costs.

When two tasks with modality-compatible mappings need to be maintained in working memory, working-memory load should be lower because both tasks can be processed more or less independently in distinct subsystems (Baddeley, 1992, 2010). For example, the auditory–vocal task requires the phonological loop and the visual-manual task requires the visuospatial sketchpad, whereas in the modality-incompatible visual–vocal task, the visual stimulus would refer to the visuospatial sketchpad, but the vocal response (and its anticipated auditory effect) to the phonological loop. Hence, with both tasks referring to distinct subsystems, between-task crosstalk should be lower (see Maquestiaux et al., 2018; Schacherer & Hazeltine, 2019).

The supplemental analysis showed that mixing costs for auditory-manual trials were particularly affected by spatial-verbal processing codes and temporal-discrimination task demands (each compared to spatial-location codes/task demands). That is, when sensory input in different modalities needs to be processed and also reactions in different modalities are required, auditory stimuli tend to require vocal responses (as it is common in conversations in everyday life) and this tendency is even increased when auditory input was also verbal in nature. This suggests that, as predicted, the auditory–vocal coupling was indeed strengthened by spatial-verbal codes. This pattern is consistent with Wickens (1984), and we can specify that the definition of compatibility of mappings can be derived from ideomotor theorizing proposing a strong role of anticipation of sensory action effects in action selection generally (Greenwald, 1972; Shin, Proctor, & Capaldi, 2010).

While there is a similar connection between spatial-location tasks and the visual-manual mapping (Wickens et al., 1984), the similarity in the present study between a manual keypress and a white diamond-shaped stimulus is obviously much smaller than between an auditory stimulus and a vocal response which both produce the exact same word. Thus, the ideomotor linkage between the spatial-location task and the visual-manual coupling should be considerably weaker than the ideomotor linkage between the spatial-verbal task and the auditory–vocal coupling. Hence, spatial-verbal stimuli can be assumed to have strengthened the auditory–vocal coupling to a greater extent than the spatial-location stimuli may have strengthened the visual-manual coupling.

### The role of processing codes in switch costs

Regarding switch costs, however, the prediction that interference due to spatial-verbal codes or temporal-duration task demands should be particularly strong on switch trials could not be confirmed: While the modality-compatibility effect was still numerically present in the expected direction for both spatial-verbal codes and temporal-duration task demands, spatial-location codes/task demands yielded a speed-accuracy trade-off that was not observed in earlier studies (Fintor, Poljac et al., 2018; Fintor, Stephan et al., 2018a, 2018b; Schacherer & Hazeltine, 2019; Stephan & Koch, 2010, 2011, 2015, 2016; Stephan, Koch, Hendler, & Huestegge, 2013). Specifically with regard to spatial-verbal codes, our results are also not completely in line with the study by Schäffner et al. (2018), who had indeed found larger switch costs with verbal compared to nonverbal stimulus material, and with incompatible modality mappings, while we found larger switch costs with spatial-location (nonverbal) stimuli and compatible modality mappings in RT, and larger switch costs for incompatible modality mappings in error rates, but with *both* types of processing codes. The important distinction though, as mentioned earlier, is that in the spatial-verbal condition in the study by Schäffner et al. (2018), only the responses actually featured a spatial component, whereas the stimuli referred to semantic categories; in our Experiment 1, both the spatial-verbal stimuli and the required responses were conceptually overlapping, since both referred to the spatial dimension.

Schacherer and Hazeltine (2019) demonstrated how differences in switch costs between compatible and incompatible modality mappings could be made to disappear by reducing conceptual overlap between the tasks. However, since our participants only switched between modality mappings, that is, there were no trial-to-trial switches between spatial-verbal codes/temporal-duration task demands and spatial-location codes/task demands, we would argue that the amount of conceptual overlap within one processing code or task demand was equal: It either always referred to a spatial code (with spatial-verbal and spatial-location stimuli) or duration (with temporal-duration). Note that in the context of dual-task training, Maquestiaux et al. (2018) explained the beneficial effects of modality compatibility in terms of Baddeley’s (e.g. 1992, 2010) model of working memory, suggesting that response selection should be easier with compatible modality mappings because both stimulus and response codes referred to the same subsystem. However, this more general approach can explain the effect on mixing costs (see previous section); it is less clear how it could explain the specifically increased between-task crosstalk in task switches relative to repetitions.

Based on our ideomotor approach, we can integrate the notion of distinct working memory subsystems, so that these are not mutually exclusive accounts, even though we believe that our account is more specific. In particular, we propose that in Experiment 1, spatial-location codes may be processed in the visuospatial sketchpad, while spatial-verbal codes would be held primarily by the phonological loop (because their “spatial” component is merely semantic; their physical location was central/binaural, i.e. neutral). Hence, it would be more difficult to select the proper response code from one working-memory subsystem if both the code for the stimulus modality and the spatial-verbal/spatial-location code are processed in the respective other subsystem. In the supplemental analyses, the auditory-manual mapping showed larger interference with spatial-verbal codes (because both the auditory stimulus and the spatial-verbal code point towards the phonological loop, while the anticipated effect of the correct manual response should be processed in the visuospatial sketchpad), whereas the visual–vocal mapping showed increased interference, if at all, with spatial-location codes (because both the visual stimulus and the spatial-location code point towards the visuospatial sketchpad, while the anticipated effect of the correct vocal response should be contained in the phonological loop). In turn, auditory-manual spatial-location trials and visual–vocal spatial-verbal trials are “easier” because two of the three relevant codes are contained in the subsystem that features the correct response (spatial-location and manual in the visuospatial sketchpad, spatial-verbal and vocal in the phonological loop). This idea could also be transferred to Experiment 2: While Baddeley (2010) described how a visual verbal stimulus can be transformed into the phonological loop like a spoken word, it is by no means obvious that an analogous translation into the duration of some type of sound would happen to a visual temporal-duration stimulus. If indeed this does not occur, it would explain why, with the visual–vocal mapping, the temporal-duration task was not sufficiently easier than the spatial-location task to yield a significant difference in switch costs, as it was found for the same modality mapping between spatial-verbal and spatial-location processing codes.

### Theoretical implications

As noted in the previous section, both multiple resource accounts (Wickens, 1984, 2008) as well as working memory subsystem accounts (Maquestiaux et al., 2018; see also Schacherer & Hazeltine, 2019) and the between-task crosstalk account that we propose in line with previous studies (e.g. Stephan & Koch, 2011, 2015, 2016) are not mutually exclusive but differ in their degree of specificity. Both could explain modality-compatibility effects in mixing costs (or dual-task costs in dual-task contexts, e.g. Göthe et al., 2016), whereas our ideomotor approach, based on anticipation of response effects (see also Wirth, Steinhauser et al., 2018), can also explain specifically increased interference in switch trials relative to repetition trials.

The present study adds to the body of evidence strengthening the claim that “central” processes, as put forward by both bottleneck accounts (e.g. Pashler, 1994) and resource-sharing approaches (e.g. Kahneman, 1973; Navon & Miller, 2002; Tombu & Jolicœur, 2003), are affected by modality-specific influences. Specifically, the increased mixing-costs with modality-incompatible mappings, as well as increased switch costs with such mappings in error rates, further strengthen the support for the between-task crosstalk account (Stephan & Koch, 2011; see also Göthe et al., 2016), in line with ideomotor approaches to action control (e.g. Greenwald, 1970, 1972; Shin et al., 2010). In contrast, modality-specific accounts that assume generally preferred mappings, such as visual-spatial-manual and auditory-verbal-vocal, meaning that these combinations of modalities and processing codes should be beneficial even in a single-task setting (Wickens, Sandry, & Vidulich, 1983; Wickens et al., 1984), would consequently expect incompatible modality mappings to lead to worse performance in general. However, as shown by the higher RT and error rates for the compatible condition in the single-task analysis of both experiments, this can be ruled out as an explanation for the influence of modality compatibility in multitasking situations. Our findings further extend previous research by demonstrating that the interference in form of between-task crosstalk can be further modulated by varying processing codes and task demands; specifically, such processing codes and task demands that strengthen the auditory–vocal coupling can further increase the already-present interference between mappings. Keep in mind that this between-mapping interference occurs in addition to the central interference in terms of general mixing costs and switch costs, which were still present with modality-compatible mappings. However, if both task sets feature incompatible modality mappings, spatial-verbal codes and temporal-duration task demands seem to interfere predominantly with the concurrent maintenance of two competing task sets in working memory, and only to a lesser extent with the updating and shifting from one task set to another, as it is required on switch trials.

In sum, our study provides further evidence for a modality-specific influence on cognitive control processes in the form of task-confusion due to crosstalk between two incompatible mappings. Our research extends the earlier work in this field by demonstrating that this influence can be further modulated by introducing both spatial-verbal processing codes and temporal-duration task demands compared to the previously employed nonverbal spatial-location paradigm. We attribute this to a strengthening of the auditory–vocal coupling, resulting from the tight ideomotor binding between audition and verbal content, as well as the modality appropriateness of audition for temporal discrimination.

## Footnotes

1 The influence of modality compatibility on switch costs has been repeatedly replicated with sample sizes of 16 participants (Stephan & Koch, 2010, 2011). Testing 24 participants allowed us to detect a large effect (d*z* = 0.8) at a significance level of *α* = 0.05 with a power of > 0.96 (Faul, Erdfelder, Lang, & Buchner, 2007).

2 We examined whether the order in which the tasks were presented—spatial-verbal vs. spatial-location in Experiment 1; temporal-duration vs. spatial-location in Experiment 2—might have influenced performance specifically in the spatial-location task. Yet, RT switch costs on the spatial-location task were consistently at least numerically larger with modality-compatible mappings, and error switch costs were larger with modality-incompatible mappings. This means the trade-off was always present, irrespective of the order in which the tasks were presented. Note that participants who started with the spatial-location task were, at the time of performing the spatial-location task, facing the same conditions as subjects in preceding studies that exclusively employed the spatial-location task (e.g. Fintor, Stephan et al., 2018a; Stephan & Koch, 2010). This leaves no obvious explanation for the trade-off in our study.
